# Retraction: Clonogenic, myogenic progenitors expressing MCAM/CD146 are incorporated as adventitial reticular cells in the microvascular compartment of human post-natal skeletal muscle

**DOI:** 10.1371/journal.pone.0304210

**Published:** 2024-05-28

**Authors:** The PLOS ONE Editors

Following publication of this article [1], concerns were raised about undeclared re-use of data from an earlier study [2] by some of the same authors.

Specifically:

The study reported in [1] appears to overlap with work reported previously in [2] (which was published under a CC BY-NC-ND 4.0 license), including in methodology, results and conclusions.

Related to the overlap in experimental work, some images in [1] are based on the same tissue/cell samples reported in [2]. The reuse of samples and/or images was not declared in [1].

- The fluorescence microscopy image in Fig 1A of [1] shows the same cell as is shown in S4E Fig panel g of [2]. Corresponding author BS has stated that these two images are separately captured images of differing magnification, showing the same tissue sample.
- The skin fibroblast image panel in Fig 3B of [1] appears similar to the cord blood image in Fig 4Ac of [2]. Corresponding author BS has stated that the image used for the cord blood panel in Fig 4Ac of [2] is incorrect, and the *PLOS ONE* figure panel is correctly labelled as skin fibroblasts.
- In Fig 4C of [1], the two image panels labelled “myoglobes and myofibers” in the second and third rows appear similar to the MU “MSCs” image panels of Fig 5Eb and 5Ea of [2], respectively. Corresponding author BS has indicated that the images used in Fig 4C of [1] are separately captured images of the same harvested Matrigel section as shown in Fig 5Ea and 5Eb of [2], with different magnifications.
- In Fig 4B of [1], the lower right-hand Matrigel image panel with scale 10μm (described in the caption as human dermal-derived fibroblasts) is incorrect and is a higher magnification image of the same tissue shown in the BM “MSCs” Matrigel image panel in Fig 5Fb of [2].
- A region on the right-hand side of the hSpectrin Laminin Dapi panel of Fig 5D of [1] reported to show myofibers of SCID/beige/CTX (cardiotoxin) injured mice, when flipped vertically and rotated appears similar to part of the lower right-hand panel of Fig 5D of [2] reported to show myofibers from SCID/mdx mice.

In response to these concerns, corresponding author BS indicated that the study reported in [2] was a preliminary approach to questions around non-canonical myogenic progenitors and that the *PLOS ONE* article [1] addressed further pending questions, offering advances including:

- A direct comparison of the explant culture vs CD-146^+^-enriched populations, in terms of myogenic potential, frequency of myogenic cells and expression of markers; and
- The finding that *in vivo* transplanted human muscle-derived CD146^+^ multi-clonal cell strain immunoreactivity of CD146 was restricted to microvascular walls of the interstitial tissue.

A member of the Editorial Board advised that there is substantial overlap in the methodology and findings presented in these two articles [1,2], including several instances of duplicated data reported from the same experiments and samples. They noted that while there are some experimental results that are newly described in the *PLOS ONE* article [1], those results are insufficient to support a standalone article if the overlapping content is disregarded.

In light of the concerns about the extent of the overlapping content and the undeclared re-use of experimental data from [2], the *PLOS ONE* Editors retract this article because it does not satisfy the journal’s second publication criterion or PLOS’ policy on Submission and Publication of Related Studies.

Of note, three additional issues arose in regard to this article [1]:

- The study approval by the Research Ethics Committee of Istituto Superiore di Sanità of Rome (approval date September 20, 2016; Prot. PRE-686/16) was incorrectly reported in this article and does not apply to the work reported in [1]. The corresponding author BS has indicated that an earlier version of the manuscript reported the human muscle samples were collected with patient consent as surgical waste following orthopedic surgery. The principal investigator for the study has passed away, and corresponding author BS has indicated that they hold no further information about ethical oversight or patient consent for the work. Hence, this issue remains unresolved.
- The article’s Funding statement as published is incorrect. The corresponding author BS has indicated that the correct statement was: The first phases of this work were supported by a Genostem grant (LHSB-CT-2003-503161) to Prof. Paolo Bianco. The funders had no role in study design, data collection and analysis, decision to publish, or preparation of the manuscript.
- There are also several references to “Data not shown” in support of statements in the originally published article, which is not permitted under *PLOS ONE*’s editorial policies. The corresponding author BS provided these data for editorial consideration, and so this issue could have been resolved.

The Fig 1A, Fig 3B skin fibroblasts panel, Fig 4C myoglobes and myofibers panels, Fig 4B lower right-hand Matrigel panel, and Fig 5D hSpectrin Laminin Dapi panel results report material similar to that published in [2] in 2016 by Sacchetti et al. under a CC BY-NC-ND 4.0 license. Due to restrictions that apply to the original article’s license, these figures are excluded from the PLOS article’s [1] CC BY 4.0 license. At the time of retraction, the article [1] was republished to note this exclusion in the Figure legends and the article’s copyright statement.

TP and MC agreed with the retraction. AF and BS either did not reply directly or could not be reached.
